# Key Applications of Plant Metabolic Engineering

**DOI:** 10.1371/journal.pbio.1001879

**Published:** 2014-06-10

**Authors:** Warren Lau, Michael A. Fischbach, Anne Osbourn, Elizabeth S. Sattely

**Affiliations:** 1 Department of Chemical Engineering, Stanford University, Stanford, California, United States of America; 2 Department of Bioengineering and Therapeutic Sciences, University of California San Francisco, San Francisco, California, United States of America; 3 Department of Metabolic Biology, John Innes Centre, Norwich Research Park, Norwich United Kingdom; The Sainsbury Laboratory, United Kingdom

## Abstract

Great strides have been made in plant metabolic engineering over the last two decades, with notable success stories including Golden rice. Here, we discuss the field's progress in addressing four long-standing challenges: creating plants that satisfy their own nitrogen requirement, so reducing or eliminating the need for nitrogen fertilizer; enhancing the nutrient content of crop plants; engineering biofuel feed stocks that harbor easy-to-access fermentable saccharides by incorporating self-destructing lignin; and increasing photosynthetic efficiency. We also look to the future at emerging areas of research in this field.

## Abstract 2

Elizabeth Sattely, Anne Osbourn, and colleagues discuss in this Essay four long-standing challenges in plant metabolic engineering: to create plants that provide their own nitrogen, have improved nutrient content, function better as biofuels, and have increased photosynthetic efficiency.

This article is part of the *PLOS Biology* Collection “The Promise of Plant Translational Research.”

## Introduction

In their native form, plants constitute a remarkable feat of metabolic engineering. Not only does their energy derive entirely from the sun and their carbon from CO_2_, but they can defend themselves from pests and predators without the benefit of mobility; they participate in complex symbioses, in part by tailoring the composition of their epi- and endophytic microbial communities, and they can survive extremes of temperature and nutrient and water availability. What more could we ask of plants?

A great deal, it turns out. Both conventional breeding and modern metabolic engineering have been used to boost productivity and to enhance fitness (for example, by increased resistance to pests, herbicides, and climatic extremes) [1]. In addition, new areas of application have been introduced that would have seemed like science fiction only a few decades ago, including the use of plants to produce vaccines, bioplastics, and derivatives of complex natural product drugs [2]–[4]. Many of these more recent engineering goals could not have been accomplished by fine-tuning endogenous host metabolism; instead, they required the installation of new metabolic pathways from other plants or bacteria. Adding new nodes to a plant metabolic network is a difficult task that will benefit from advances in targeted genome modification, tissue-, cell-, and organelle-specific gene expression, and the controlled expression of multi-gene pathways [5]–[7].

In this essay, we highlight recent progress in, and the near-term potential of, four long-standing grand challenges in plant metabolic engineering: two deal with important applications in food and energy, while the remaining two are of general utility in improving plant fitness, and in principle would be useful for improving plants as a chassis for other metabolic engineering efforts. Nature never intended plants to be grown as crops on an industrial scale, nor did plants evolve solely for human nourishment. Plants are not naturally inclined to give up their structural oligosaccharides in ready-to-eat form in the service of providing green energy. Although each of these challenges has been recognized for decades and important advances have been made [8]–[10], solutions to them still lie far beyond our current capabilities. Nevertheless, the technologies developed to meet them will have myriad uses long before the problems themselves are solved. Techniques for using synthetic biology to make multiple deletions, additions, and other edits to plant genomes stand out as a particularly important set of enabling technologies for the challenges described below [11]. Finally, while we focus primarily on the technical aspects involved in developing these engineering efforts, we recognize that addressing societal acceptance, economic considerations, environmental impact, and long-term sustainability are also of critical importance for their successful implementation.

## Plants That Can Fix Their Own Nitrogen

A remarkable 180 million tons of nitrogen fertilizer is used every year in industrial farming [12], and it has played such an enabling role that fertilizer use is estimated to meet the nutritional needs of one-third of the human population [13]. However, the use of nitrogen fertilizers has serious disadvantages, including substantial cost and deleterious effects on the soil and surrounding environment, and neighboring waterways. Plants that could satisfy some or all of their own nitrogen requirement would transform agriculture by reducing or eliminating this enormous dependence on fertilizer.

There are two conceivable ways in which a plant could be engineered to satisfy its own nitrogen requirement. The first takes advantage of the fact that some bacteria carry out their own version of the Haber-Bosch process—reducing atmospheric N_2_ into a more bioavailable form, NH_3_—using the enzyme nitrogenase [14]. This complex enzyme contains multiple metalloclusters and requires a large quantity of biochemical energy to transfer the electrons needed to activate the exceptionally stable N_2_ triple bond. By expressing nitrogenase, plants would be able to fix their own nitrogen. The primary advantage of this approach is that it is direct: nitrogen fixed by a plant could be used immediately to generate amino acid and nucleic acid monomers and transport them to neighboring cells. Although the process would incur a metabolic cost, it could be regulated by the endogenous level of nitrogen to maximize its efficiency.

Immense technological challenges stand in the way of accomplishing this goal [15]. Eighteen gene products (in *Klebsiella oxytoca*) are necessary and sufficient for the production of nitrogenase and its complex iron-molybdenum cofactor. By an impressive feat of microbial engineering, the biosynthetic gene cluster for nitrogenase has been refactored—taken apart, re-coded, and put back together using known components—and shown to be active in its new host [16]. The successful transfer of other large gene clusters from one microbe to another, such as the one encoding the magnetosome, suggests that the process of functionalizing microbes is undergoing a dramatic improvement [17].

But new challenges must be overcome for the expression of similar elements in plants. First, all 18 components of the nitrogenase biosynthetic apparatus would need to be expressed simultaneously in plants and function in concert, a considerable barrier given that the largest number of genes expressed in an engineered plant to date is eight in the establishment of a photorespiratory bypass in *Arabidopsis* [18]. Second, since plants are eukaryotic and multicellular, where in the plant cell should the genes be expressed and in which cell types of the plant? This question is especially relevant for nitrogenase, which is poisoned by oxygen and must therefore be expressed under anaerobic conditions. Tools that enable organelle- and cell-type specific expression will be of great utility here and in other plant engineering efforts.

The second way to reduce or eliminate the need for nitrogen fertilizer would be to engineer a rhizosphere symbiosis between a nitrogen-fixing microorganism and a plant host. Although this approach is less direct than expressing nitrogenase in plants, it has two primary advantages: (1) It uncouples the difficulties of utilizing nitrogenase (e.g., sequestering the enzyme in an anaerobic compartment) from the biology of the plant host, and outsources the demanding chemistry involved to a bacterial strain better suited to the task. (2) The well-studied symbioses between legumes and their nitrogen-fixing, root-nodulating bacterial symbionts prove that a bacterial mutualist can satisfy the nitrogen needs of a plant host [19]. Even though root-nodulating bacteria appear to be specific to legumes, the presence of nitrogen-fixing bacteria in the rhizosphere opens the possibility that symbioses of this sort are a much more widely distributed phenomenon [20]. If so, then the feasibility of making this mode of nitrogen exchange more efficient—rather than engineering it from scratch—would appear high. However, there remain two primary challenges in engineering a stable and practical rhizosphere symbiosis: enabling efficient nutrient exchange and maintaining specificity of the host-microbe pair. Both could take years to develop and are likely to require not just plant but also microbial metabolic engineering. In the meantime, advanced molecular breeding tools that enable access to natural variation in a plant's wild ancestors [21] are a promising alternative approach to increasing crop plant yields [22].

## Crop Plants with Altered Nutrient Content

What if maize could be as nutritious as broccoli, and broccoli as palatable as maize [23]? Golden rice proves the concept that the nutrient content of a crop plant can be improved by metabolic engineering [24]. Centuries of breeding not just rice but maize, wheat, tomatoes, and cruciferous vegetables have undoubtedly enhanced the levels of certain nutrients while suppressing others [25]. In the long run, adding the beta-carotene pathway to rice, to produce rice with higher levels of vitamin A (yielding the strain known as Golden rice), may prove to be among the more modest changes to nutrient content that are achievable by metabolic engineering.

One class of targets for nutrient engineering are the metabolic pathways that produce phytoalexins, flavonoids, and other molecules that are thought to play a role in the chemopreventive properties of vegetables and fruits. One approach is to alter the level of a nutrient in its native host, a strategy that does not require knowing the genes in its biosynthetic pathway. Two examples illustrate the feasibility of this approach. In the first, a tour de force in conventional plant breeding led to the Beneforte strain of broccoli, which has higher levels of the glucosinolate glucoraphanin than traditional cultivars of broccoli (and is already being sold at some US grocery stores) [26]. Early studies have demonstrated that human consumption of high-glucoraphanin broccoli results in improved metabolism and reduced levels of fatty acids and in the lipid compounds associated with inflammation. In the second example, by expressing two transcription factors from snapdragon in tomato, the levels of the flavonoid anthocyanin have been increased 3-fold, a level that was sufficient for this flavonoid to confer improved chemopreventive properties in cancer-susceptible mice [27].

A different approach would be to express the pathway for a health-promoting molecule in a new host. Golden rice provides one example of this strategy, albeit a simple one from the viewpoint of genetic engineering since only two genes were required for the production of beta-carotene (although beta-carotene is an endogenous metabolite, it is not produced in the endosperm, the edible portion of the plant) [28]. A more ambitious prospect would be to transfer the 13-gene glucoraphanin pathway to a widely consumed crop such as rice, wheat, or maize [29]. Such an effort would require new approaches for discovering the genes for plant nutrient biosynthetic pathways. In addition, new tools for site-selective genome editing would be needed to express multiple enzymes in a pathway or multiple pathways in a plant [30]. By analogy to previous efforts in bacterial metabolic engineering, additional genetic changes might be required to boost the levels of key precursors to the molecule of interest [31].

Introducing or increasing the level of a nutrient compound could also alter the taste of a plant, potentially impacting its palatability. Likewise, the challenge of improving the taste profile of a plant could be addressed by a similar strategy, since small molecule metabolites make an important contribution to flavor. Notable targets in this area include the steviol glycosides and the mixed esters that give strawberry plants their distinctive flavor [32].

## Engineering Crops for Biofuel Production

Plants would seem to be the ideal “invention” to combat the dual challenges of rising greenhouse gases and the need for green energy: they capture CO_2_ from the air and turn it into sugar, the ideal substrate for biofuel production. Not surprisingly, though, plants protect this energy rich polymer—most of the carbon is stored as dehydrated crystalline cellulose, wrapped in a meshwork of crosslinked phenylpropanoids, lignin [33]. In solving the problem of how to create a rigid skeleton that enables a sessile organism to resist pests, pathogens, wind, precipitation, and temperature extremes, plants have managed to sequester their carbon in a form that is complicated to unwrap.

Cellulose presents a challenge in itself. Other polymers of glucose, including starch and glycogen, are rich energy sources that are easily degraded into sugar monomers. The beta-1,4-linked chains of cellulose, on the other hand, can pack tightly together, excluding water in a way that prevents glycosidic enzymes from releasing its constituent sugar monomers. On top of the challenge of hydrolyzing cellulose, the lignin with which it co-purifies can inhibit the action of cellulases, creating a need for costly and energy-intensive pretreatment to separate the cellulose from lignin [34].

Strategies have been proposed in which the lignin could be degraded into valuable aromatic monomers either chemically or by enzymes found in white rot fungi, microorganisms that naturally degrade lignin [35],[36]. Both remain exciting prospects, although neither has been shown to work in a real-world setting.

Alternatively, lignin biosynthesis can be genetically modified to change its chemical composition or to reduce its content in plant tissues to improve the processing of polysaccharides. For example, an *Arabidopsis thaliana* knockout mutant of caffeoyl shikimate esterase, a newly discovered lignin biosynthetic gene, gave a 4-fold increase in the efficiency of saccharification—the breakdown of oligosaccharides to monosaccharides—relative to wild-type [37]. However, typical of many lignin-modified plants [38], there were discernible effects on plant growth and development; the transgenics contained ∼25% less cellulose content and were ∼40% lighter and smaller than wild-type. In another recent attempt, protein engineering and expression of a 4-*O*-methyltransferase in *A. thaliana* substantially reduced lignin content by blocking access to *p*-hydroxyls of lignin precursors needed for polymerization [39]. Interestingly, no significant changes in growth phenotype were observed and saccharification yields improved by ∼25%. Further success with lignin modifications to plants will depend heavily on an improved understanding of lignin biosynthesis and of the physiological consequences of altering its structure.

But what if plants did the difficult work on their own, without the need for large-scale reactors or any human intervention? Following the harvest of a biofuel feedstock, a crop grown for biofuel production, the ideal scenario would be for plants to degrade their own lignin, releasing pure cellulose that could be more easily degraded into glucose. For that matter, the plant could be engineered to break down its own cellulose on demand, releasing fermentation-ready sugars for biofuel production.

Two formidable challenges stand in the way of realizing this goal. The first is the feasibility of enzymatically degrading lignin to liberate cellulose. Although this would undoubtedly be a difficult task, the ability of white rot fungi to degrade lignin proves the concept that there exist enzymes (e.g., lignin peroxidases) that can cleave the lignin meshwork into monomers and smaller polymers [40]. Nor is it necessary for the lignin to be degraded; an alternative possibility would be to more heavily crosslink (and consolidate) the lignin, causing it to precipitate and making it easier to separate from cellulose. Since this is a process that will likely be carried out by suites of degrading enzymes in rot fungi, a critical step would be to first identify sets of enzymes that could be co-expressed to make the necessary modifications to lignin.

An additional advance would be to regulate the production of such enzymes with exquisite specificity. Since transcription and translation are unlikely to persist long after harvest, the enzymes could instead be expressed in an inactive, caged form while the plant is still alive. Any small amount of leaky activity prior to harvest could compromise the plant structurally, or make it susceptible to pathogens and pests. Too little activity, on the other hand, would fail to release the cellulose from lignin. One informative model is that of myrosinases, enzymes in cruciferous plants that activate glucosinolates, a family of defense compounds, by glycoside hydrolysis [41]. These enzymes are translated but physically sequestered from their glucosinolate substrates in different cells, activated only upon injury of the plant tissue. Physical sequestration of the constituent enzymes in a “degradome” could likewise serve to cage their activity until after harvest. A recent paper reports a promising alternative strategy based on incorporating chemically labile bonds into the lignin backbone [42].

## Enhancing Photosynthetic Efficiency

As the world population and urbanization rapidly increase, increases in crop yield will be required to meet growing food demands. Cereals will continue to be an especially important part of the future food supply both for direct consumption and as livestock feed. Crop yields for biofuel feedstocks will also need to be considered for green energy production.

Rubisco is the enzyme that catalyzes the first key step in CO_2_ fixation as part of the Calvin Cycle [43]. Its low turnover rate and ability to also use oxygen as a substrate in photorespiration make it notoriously inefficient. As a result, plants make more Rubisco than any other protein, making it the most abundant protein in the world.

Alternative carbon fixation systems have evolved to improve the efficiency of photosynthesis by actively concentrating CO_2_ and reducing the oxygenase activity of Rubisco [44],[45]. In plants, the two systems that have evolved to improve photosynthesis efficiency are C4 and Crassulacean acid metabolism (CAM) photosynthesis. Unlike in C3 plants (e.g., rice and other cool-season cereals), in which the process of fixing carbon into C3 acids occurs in the same cell type, C4 plants have evolved to separate the Calvin cycle and carbon capture into different cell types. CO_2_ is first captured within mesophyll cells to produce C4 acids, which diffuse to bundle sheath cells where they are decarboxylated and concentrated to maximize Rubisco's carboxylating efficiency. In CAM plants, photosynthesis and carbon capture are separated temporally instead of spatially. Living in arid conditions, CAM plants capture CO_2_ at night into C4 acids, enabling them to close their stomata during the day to prevent water loss. As with C4 photosynthesis, the C4 acids generated by CAM photosynthesis are decarboxylated and concentrated to enhance Rubisco's efficiency.

C4 plants generally have better nitrogen and water-use efficiencies than C3, particularly in hot and dry climates. C4 plants also have radiation use efficiencies—the ability to convert photons into biomass—that are 50% greater than those of C3 plants, which has led many to suggest that engineering C3 plants into C4 could potentially lead to a 50% increase in crop yield. The International Rice Research Institute is currently working toward incorporating C4 photosynthesis into rice (http://c4rice.irri.org/).

The evolution of C3 to C4 plants has occurred independently in more than 60 different taxa, indicating that this engineering effort may be more feasible than it might otherwise appear, but many new plant metabolic engineering technologies will be required [46]. All the enzymes of the C4 cycle are known and already exist in C3 plants. However, expressing the enzymes of the C4 cycle alone will not be enough, as the plant's anatomy is crucial for the success of the pathway. The genes that are responsible for controlling C4 leaf anatomy remain largely unknown and are being identified by mutant populations of model C4 plants like Sorghum [47]. Additionally, cell-specific promoters will need to be identified to enable cell-type-specific expression in bundle-sheath or mesophyll cells. Finally, with >20 genes needed for the installation of C4 photosynthesis in C3 plants, the development of more sophisticated transformation and genome editing technologies will be required.

## Conclusion

The long-standing nature of these challenges highlights two needs: First, this field would benefit tremendously from increased funding, especially from federal agencies that have not traditionally invested in plant biology. The likely impact of plant metabolic engineering on the future of fuel and food suggests that funding agencies focused on human health and energy security should consider plant metabolic engineering a priority. Initiatives that encourage the training of plant metabolic engineers—people who understand basic plant biology and fundamental principles of engineering—are especially critical to the future success of the field.

Second, the design-build-test cycle in plant engineering needs to be accelerated. Three classes of technologies will be of particular importance: (i) Transcriptomic [48] and metabolomic [49] analyses are capable of rapidly generating functional data that inform engineering efforts, especially in non-model hosts. Computational analyses that glean insights from these data to predict genes of importance for, e.g., nitrogen utilization, will be particularly enabling. (ii) Genome editing tools will enable multiple changes to be made simultaneously to a broad range of model- and non-model plants. Recent advances in using TALENs [50] and CRISPR/Cas9 [51] to engineer plant genomes hold unusual promise in launching far more ambitious efforts to systematically engineer plants. (iii) A larger synthetic biology parts list specific to plants that includes tissue-specific promoters, transporters, multi-gene expression constructs, and biosynthetic enzymes (for example, see [52]). Taken together, these technologies would enable the manipulation of plant metabolism at an unprecedented level, and promise to translate basic knowledge of plant metabolism into tangible benefits for agriculture.

## Abbreviations

CAM: Crassulacean acid metabolism
